# Risk factors for cardiovascular disease and type 2 diabetes retained from childhood to adulthood predict adult outcomes: the Princeton LRC Follow-up Study

**DOI:** 10.1186/1687-9856-2012-6

**Published:** 2012-04-16

**Authors:** John A Morrison, Charles J Glueck, Jessica G Woo, Ping Wang

**Affiliations:** 1 From the Division of Cardiology, Children’s Hospital of Cincinnati, 3333 Burnet Avenue, 45229, Cincinnati, USA; 2 From the Cholesterol and Metabolism Center, Jewish Hospital of Cincinnati, Cincinnati, USA; 3 Cholesterol Center, UC Health Business Center, 3200 Burnet Avenue, Cincinnati, OH 45229, USA

**Keywords:** Risk factors, Cardiovascular disease, Type 2 diabetes mellitus, Obesity, High blood pressure, Tracking

## Abstract

**Background:**

Pediatric risk factors predict adult cardiovascular disease (CVD) and type 2 diabetes (T2DM), but whether they predict events independently of adult risk factors is not fully known.

**Objective:**

Assess whether risk factors for CVD and T2DM retained from childhood to adulthood predict CVD and T2DM in young adulthood.

**Study design:**

770 schoolchildren, ages 5–20 (mean age 12), 26-yr prospective follow-up. We categorized childhood and adult risk factors and 26-year changes (triglycerides [TG], LDL cholesterol, BMI, blood pressure [BP] and glucose ≥, and HDL cholesterol < pediatric and young adult cutoffs). These risk factors and race, cigarette smoking, and family history of CVD and T2DM were assessed as predictors of CVD and T2DM at mean age 38.

**Results:**

Children who had high TG and retained high TG as adults had increased CVD events as adults (*p* = .0005). Children who had normal BMI and retained normal BMI as adults had reduced CVD events as adults (*p* = .02). Children who had high BP or high TG and retained these as adults had increased T2DM as adults (*p* = .0006, *p* = .003).

**Conclusions:**

Risk factors for CVD and T2DM retained from childhood to adulthood predict CVD and T2DM in young adulthood and support universal childhood screening.

## Background

Recently, an expert panel recommended screening all American children for risk factors for cardiovascular disease (CVD) [1], not just children of parents with known CVD or high risk factors. Pediatric risk factors for atherosclerosis have been shown to associate with young adult atherosclerotic lesions [2], carotid intima-media thickening (CIMT) [3-7], and cardiovascular disease (CVD) events [8]. Moreover, adolescent CVD risk factor status predicts increased adult CIMT, independent of adult risk factors [9,10], and children with metabolic syndrome (MetS) have 2–3 times greater risk of high CIMT and type 2 diabetes mellitus (T2DM) as adults versus children free of MetS [11]. Obese children who remained obese as adults have increased type 2 diabetes (T2DM), hypertension, dyslipidemia, and increased CIMT, but risks for these outcomes are attenuated in obese children who became non-obese adults, with outcomes similar to those non-obese in both childhood and adulthood [12].

It is not well understood whether childhood risk factors cause adult CVD directly, independent of adult risk factors, or only in those individuals whose risk factors tracked into adulthood, thereby increasing the length of exposure to high risk factors [10,13]. The best predictor of high blood pressure (HBP) in adulthood has been reported to be adult obesity or change in obesity; pediatric obesity does not predict adult HBP when adult obesity is in the model [13]. However, HBP is itself a risk factor, not a CVD event. Positive associations between childhood BMI and adult CIMT are generally attenuated once adjusted for adult BMI [14]. Child-adult relationships may be dependent on tracking of BMI from childhood to adulthood [14], since risk of events increases with the length of exposure [15,16]. There is a stepwise increase in the incidence of T2DM with the duration of obesity [15].

In the current study, our specific aim was to assess whether risk factors for CVD and T2DM retained from childhood to adulthood predict CVD and T2DM in young adulthood or whether childhood risk factors are attenuated [12] by changes in risk factors from childhood to young adulthood.

## Methods

We used data from the NHLBI Princeton Follow-up Study (PFS, 1999–2003), a 22–30 year follow-up of black and white former schoolchildren first studied in the NHLBI Lipid Research Clinics (LRC, 1973–1976) [8,17]. PFS collected data following a protocol approved by the Children’s Hospital Institutional Review Board, with signed informed consent [17].

### Princeton LRC and PFS studies

The Princeton LRC [18] study was a multistage survey of lipids and other CVD risk factors in students in grades 1–12 and a 50% random sample of their parents by household. The student population in LRC was 72% white and 28% black, with a mean age of 12.3 ± 3.4 years. Participation rates did not differ significantly between races.

The PFS [8] was conducted in adults, 22 to 30 years after their initial pediatric LRC sampling to assess relationships of pediatric risk factors to subsequent adult health events. The subjects’ CVD, T2DM and high blood pressure (HBP) status and use of prescribed medications for lipids, diabetes mellitus, and blood pressure were obtained by questionnaire with an interviewer [8].

After an overnight fast, blood was drawn for measurements of plasma triglyceride (TG), high density lipoprotein cholesterol (HDLC), low density lipoprotein cholesterol (LDLC), systolic blood pressure (SBP), diastolic blood pressure (DBP), BMI, and glucose in children and their parents at the LRC assessment and at the subsequent PFS study 26 years later. There was no contact with the former schoolchildren or their parents during the 26-year interval between the LRC and PFS studies.

### Diagnosis of CVD, type 2 diabetes and impaired fasting glucose

At PFS, CVD was defined as myocardial infarction, coronary artery bypass graft, angioplasty, ischemic stroke, and carotid or peripheral artery bypass surgery [8]. Diagnosis of diabetes (T2DM) was based on World Organization of Health criteria, fasting glucose ≥ 7 mmol/l (126 mg/dl) and/or self-report of diabetes with treatment by a physician [19]. We excluded from these analyses 10 subjects who had reported type 1 diabetes mellitus as children at LRC. However, in PFS we did not have a measurement of C-peptides or diabetes autoantibody levels, the gold standard methods of distinguishing type 1 from type 2 diabetes [19]. Diagnosis of impaired fasting glucose (IFG) was made when fasting blood glucose was ≥ 100 but <126 mg/dl.

### Pediatric and young adult risk factor cutoffs

Pediatric risk factor cutoffs included high LDLC (≥110 mg/dl [2.82 mmol/l]) [20], high BMI (≥85^th^ CDC 2000 age-gender specific percentile), high BP (SBP and/or DBP >90^th^ age-height specific percentiles in current cohort), and cutoffs published for pediatric metabolic syndrome [21]: high TG (≥110 mg/dl [1.24 mmol/l]), low HDLC (≤50 mg/dl [1.28 mmol/l] in girls, ≤40 [1.03 mmol/l] in boys), and high glucose (≥100 mg/dl [5.6 mmol/l]).

Risk factor cutoffs at the PFS were those of the NCEP/AHA Metabolic syndrome (waist ≥ 102 cm men, ≥ 88 cm women, SBP ≥ 130 mmHg and/or DBP ≥ 85, TG ≥ 150 mg/dl [1.69 mmol/l], HDLC <40 mg/dl [1.03 mmol/l] men, <50 mg/dl [1.28 mmol/l] women, glucose ≥ 100 mg/dl [5.6 mmol/l]) [22]. BMI and LDLC cutpoints at the PFS respectively were ≥ 30 kg/m^2^ (CDC, US Obesity Trends, Trends by State 1985–2009), and the current cohort’s gender-race-specific 90^th^ percentile levels.

### Statistical methods

CVD risk factor measures in the cohort in childhood (LRC) and adulthood (PFS) were summarized.

To assess for possible selection bias, comparisons were made between the 770 subjects with complete CVD risk factor measures at both the LRC visit and the PFS visit 26 years later, and 695 subjects without complete measures. Comparisons were made by Wilcoxon test or by chi-square test.

Risk factor measures at LRC and PFS were categorized as high vs. not high (for TG, LDLC, BMI, SBP-DBP, and glucose) or low vs. not low (for HDLC) using the above-mentioned cutoffs. The change in status for each risk factor from LRC to PFS was indicated by 4 dummy variables (normal to normal vs. others; abnormal to normal vs. others; normal to abnormal vs. others; abnormal to abnormal vs. others).

Stepwise logistic regression analysis was used to identify significant independent risk factors for young adult CVD, T2DM and IFG at PFS in multivariate analyses. Explanatory variables included age at follow-up and categorical variables: race, pediatric and young adult risk factor status group (high vs. not high) for TG, LDLC, BMI, glucose, blood pressure, HDLC (low vs. not low), cigarette smoking (yes vs. no), and parental history (yes vs. no) of CVD or T2DM, as well as changes in risk factor status from childhood to young adulthood. Including only significant explanatory variables from stepwise selection, logistic regression models were re-evaluated allowing more observations to be used.

From the logistic regression model, the changes in TG, BMI or BP from LRC to PFS were significant predictors for the CVD or T2DM at PFS. The associations of these risk factor changes with CVD or T2DM status were graphed. Pediatric to adult changes in risk factors were ordered to represent increasing risk, with lowest risk being normal to normal, then abnormal to normal, normal to abnormal and highest being abnormal to abnormal. The Mantel-Haenszel test was used to measure the significance of associations between the risk factor status groups in childhood and adulthood and the adult development of CVD or T2DM.

## Results

In the LRC study, there were 1465 subjects eligible for the PFS, and 909 subjects were studied in PFS 26 years later (follow-up rate 62%). Of these 909 subjects, complete data (required for the resultant logistic model) was available in 770 (85%), Table 1. Compared with the 695 eligible subjects not included in the current report, BMI was higher in the sampled group (20.1 ± 4.3 vs 19.2 ± 4.2 kg/m^2^, *p* < .0001), but there were no differences (p > 0.05) in TG, HDLC, LDLC, SBP and DBP at the LRC visit. In the sampled group, percent white was higher 73% vs 66% (*p* = .008), and percent male was lower 46% vs 57% (*p* < .0001).

**Table 1:** Risk factors for cardiovascular disease and type 2 diabetes mellitus, measured during childhood (LRC) and 26 years later in young adulthood (PFS) in 770 subjects

**Race**	W 561 (73%), B 209 (27%)				
**Gender**	M 351 (46%), F 419 (54%)				
	**At LRC**	**At PFS**	**Spearman correlationBetween LRC and PFS**		
	**Mean ± SD**	**Mean ± SD**			
Age (yr)	12.4 ±3.3	38.5 ±3.6	**Adjusted for BMI at LRC**	**Adjusted for BMI at PFS**	**Adjusted for BMI at LRC and at PFS**
	range [5–20.5]	range [29–48]			
BMI (kg/m2)	20.1 ±4.3	28.6 ±6.7			
TG (mg/dl)	77 ±38	136 ±133	r = 0.33, *p* < .0001	r = 0.34, *p* < .0001	r = 0.38, *p* < .0001
HDLC (mg/dl)	55 ±12	46 ±15	r = 0.44, *p* < .0001	r = 0.44, *p* < .0001	r = 0.46, *p* < .0001
LDLC (mg/dl)	107 ±30 106 ±29*	121 ±36 121 ±36*	r = 0.48, *p* < .0001 r = 0.49, *p* < .0001*	r = 0.48, *p* < .0001 r = 0.50, *p* < .0001*	r = 0.48, *p* < .0001 r = 0.50, *p* < .0001*
LDLC/HDLC	2.05 ±0.76 2.03 ±0.75*	2.94 ±1.34 2.94 ±1.33*	r = 0.44, *p* < .0001 r = 0.44, *p* < .0001*	r = 0.45, *p* < .0001 r = 0.45, *p* < .0001*	r = 0.46, *p* < .0001 r = 0.46, *p* < .0001*
Glucose (mg/dl)	86 ±8	90 ±23	r = 0.18, *p* < .0001	r = 0.18, *p* < .0001	r = 0.18, *p* < .0001

*After removal of 26 subjects taking cholesterol lowering medications at PFS.

Pediatric and adult CVD risk factor measures 26 years later were highly correlated, Table 1. Twenty-six of the 770 subjects (3.4%) were taking cholesterol-lowering medications at their PFS visit, Table 1. Excluding their LDLC values from the analyses of correlations between LRC and PFS did not appreciably affect the correlation coefficients, Table 1. After adjusting for BMI at mean ages 12 and 38, age 12 and age 38 risk factors remained closely correlated, Table 1.

**Figure 1:**
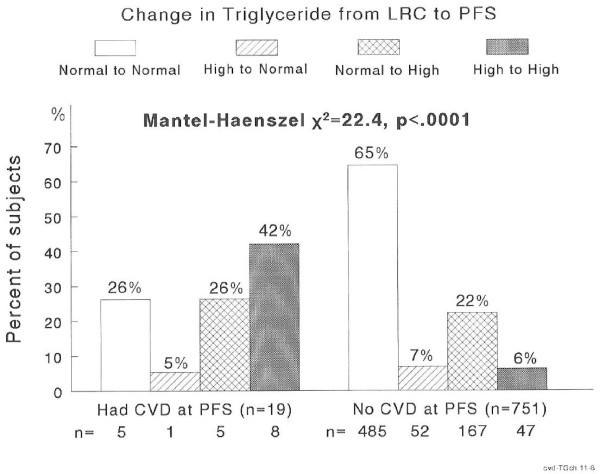
Changes in TG status groups from childhood to adulthood by adult CVD status.

High TG in childhood retained into adulthood characterized the 19 subjects who had CVD in adulthood, while normal TG at both visits characterized the 751 subjects free of CVD at the PFS visit, Figure 1. There were 55 subjects with high TG at both visits, of whom 8 (14.6%) had CVD compared to 5 of 490 with normal TG at both visits (1%) for a risk ratio of 14.6 to 1, Figure 1, Table 2. The incidence of CVD was 1.9% in subjects with high TG at LRC but normal TG at PFS, and 2.9% in subjects with normal TG at LRC but high TG at PFS. Thus, there was a linear trend for CVD across the four TG classification groups (*p* < .0001), Table 2.

**Table 2:** Incidence rate (%) of cardiovascular disease (CVD) by triglyceride (TG) and BMI classification in childhood and adulthood and of type 2 diabetes mellitus (T2DM) by blood pressure and triglyceride classification in childhood and adulthood

**Incidence rate**	**Normal - Normal**	**High - Normal**	**Normal - High**	**High - High**	**Trend of incidence rate by Mantel-Haenszel *χ*2 test**
CVD (TG)	1.0	1.9	2.9	14.6	*χ*^2^ = 22.4, *p* < .0001
CVD (BMI)	0.9	2.5	4.0	6.2	*χ*^2^ = 12.1, *p* = .0005
T2DM (BP)	3.6	4.6	8.6	28.6	*χ*^2^ = 17.8, *p* < .0001
T2DM (TG)	3.8	0	9.6	27.3	*χ*^2^ = 20.3, *p* < .0001

**Figure 2:**
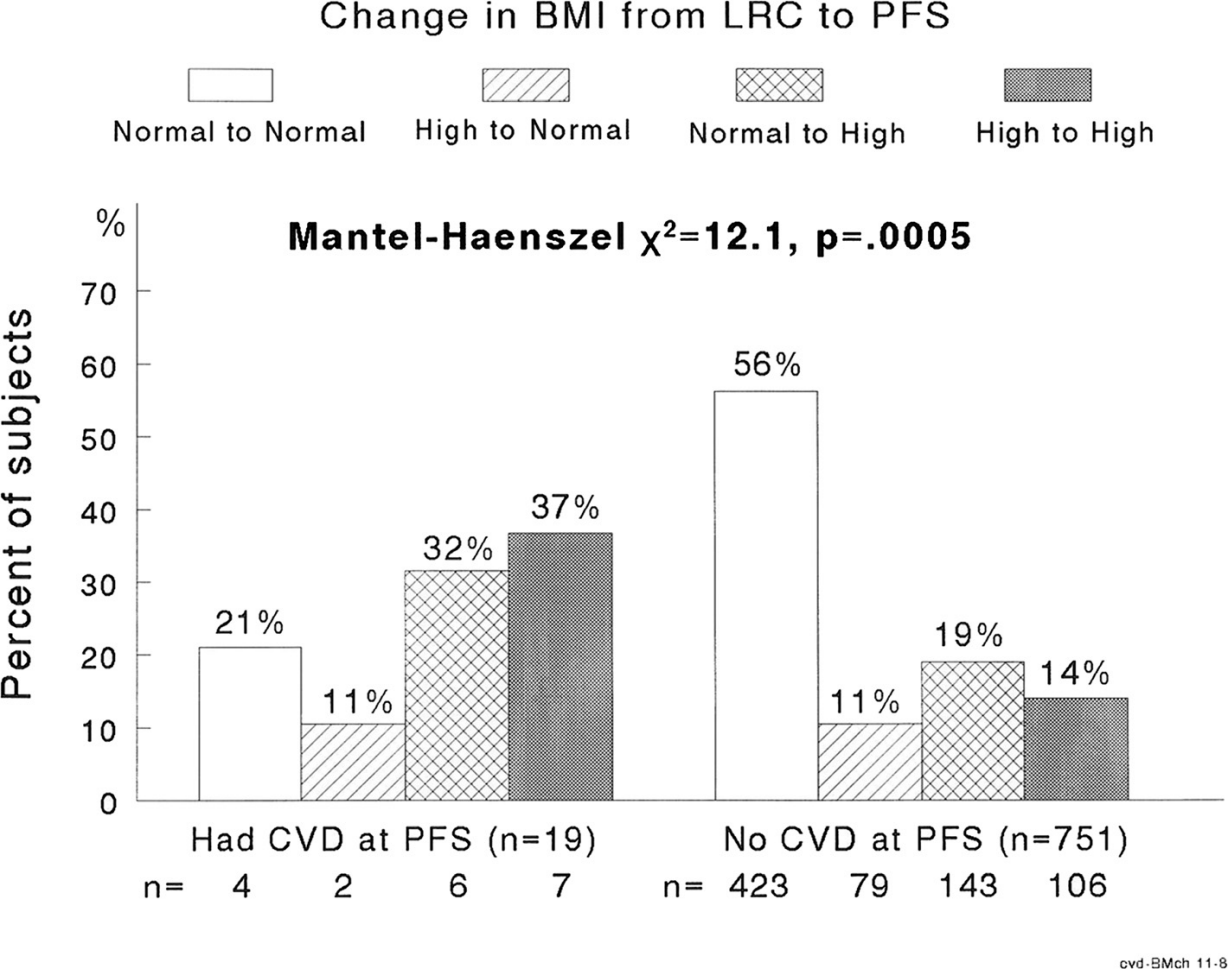
Changes in BMI status groups from childhood to adulthood by adult CVD status.

Normal BMI in childhood retained into adulthood characterized the 751 subjects free of CVD at the PFS visit, while high BMI in childhood retained into adulthood characterized the 19 subjects who had adult CVD, Figure 2. There were 113 subjects with high BMI at both visits, of whom 7 (6.2%) had CVD and 427 with normal BMI at both visits of whom 4 had CVD (0.9%) for a risk ratio of 6.9 to 1. The incidence of CVD was 2.5% in subjects with high BMI at LRC but normal BMI at PFS, and 4.0% in subjects with normal BMI at LRC but high TG at PFS. Thus, there was a linear trend for CVD across the four BMI classification groups (*p* = .0005), 2.

**Figure 3:**
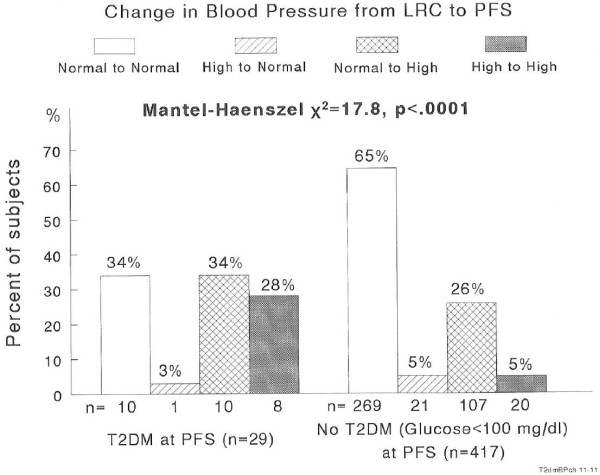
Changes in BP status groups from childhood to adulthood by adult T2DM status.

High blood pressure retained from childhood to adulthood was much more common in the 29 subjects who had adult T2DM (28%) than in the 417 free of adult T2DM (5%), while normal childhood blood pressure retained into adulthood characterized the 417 subjects free of T2DM at the PFS visit (65%), Figure 3. The incidence of T2DM was 28.6% in subjects with high BP at LRC and PFS compared to 3.6% in subjects with normal BP at both LRC and PFS, for a relative risk of 8 to 1. The one of 22 subjects with high BP at LRC but normal BP at PFS did have T2DM so the incidence rate was 4.6%. In subjects with normal BP at LRC but high BP at PFS the incidence of T2DM was 8.6%. Thus, there was a linear trend for T2DM across the four BP classification groups (*p* < .0001), Table 2.

**Figure 4:**
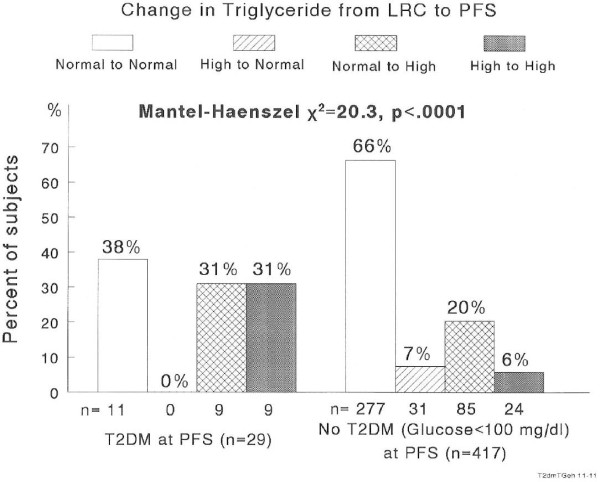
Changes in TG status groups from childhood to adulthood by adult T2DM status.

High TG retained from childhood to adulthood was much more common in subjects who developed T2DM as adults (31%) than in those free of T2DM (6%), Figure 4. The incidence of T2DM was 27.3% in subjects with high TG at LRC and PFS compared to 3.8% in subjects with normal TG at both LRC and PFS, for a relative risk of 7.1. No person with high TG at LRC but normal TG at PFS had T2DM, incidence rate = 0.0%. In subjects with normal TG at LRC but high TG at PFS the incidence of T2DM was 9.6%. Thus, there was a linear trend for T2DM across the four TG change classification groups (*p* < .0001), Table 2.

By stepwise logistic regression, adult CVD (19 yes, 751 no) was independently and significantly associated with high childhood TG retained adulthood (*p* = .0005) and with age at follow-up (*p* = .0009), and was inversely associated with normal BMI from childhood retained into adulthood, *p* = .02, Table 3.

**Table 3:** Childhood (LRC) and adulthood (PFS) predictors for cardiovascular disease (CVD), type 2 diabetes (T2DM) and impaired fasting glucose (IFG) at PFS

**Young adult outcome**	**Childhood Predictors**	**p**	**Odds Ratio, 95% Confidence Intervals**
**CVD (19 Yes, 751 no)**^**a **^**770 observations used AUC = 0.843**	TG at LRC and PFS (high to high vs others)	.0005	6.06, 2.20–16.7
	Age at PFS (year)	.0009	1.30, 1.11–1.53
	BMI at LRC and PFS (low to low vs others)	.019	0.25, 0.077–0.79
**T2DM (29 Yes, 417 normal)**^**b **^**446 observations used AUC = 0.842**	BP at LRC and PFS (high to high vs others)	.0006	6.74, 2.26–20.07
	TG at LRC and PFS (high to high vs others)	.0026	4.95, 1.75–14.06
	BMI at PFS (high vs not high)	.0010	4.56, 1.85–11.23
	Glucose at LRC (high vs not high)	.0057	5.93, 1.68–20.95
	Age at PFS	.0016	1.26, 1.09–1.45
	Black race	.043	2.53, 1.03–6.24
**IFG (88 Yes, 617 normal)**^**b **^**705 observations used AUC = 0.699**	BP at PFS (high vs not high)	<.0001	2.63, 1.64–4.23
	TG at PFS (high vs not high)	.0009	2.24, 1.39–3.60
	Cigarette smoking (yes vs no)	.018	1.78, 1.10–2.89

^a^ Logistic regression model fit after stepwise selection (SLE = .15, SLS = .05) from categorical explanatory variables: race, risk factors at LRC and at PFS (TG, HDLC, LDLC, BMI, BP and glucose), changes of risk factors from LRC to PFS (TG, HDLC, LDLC, BMI, BP and glucose), cigarette smoking (yes/no), parents had CVD (yes/no), parents had CVD before age 50 (yes/no), and parents had CVD before age 60 (yes/no), and age at PFS.

^b^ Logistic regression model fit after stepwise selection (SLE = .15, SLS = .05) from categorical explanatory variables: race, risk factors at LRC (TG, HDLC, LDLC, BMI, BP and glucose), risk factors at PFS (TG, HDLC, LDLC and BMI), changes of risk factors from LRC to PFS (TG, HDLC, LDLC, BMI and BP), cigarette smoking (yes/no), parents had T2DM (yes/no), and age at PFS.

Adult T2DM (29 yes, 417 no) was associated with BP and TG high in childhood and retained into adulthood (*p* = .0006, .003), with childhood glucose (*p* = .006), with adult age (*p* = .002), and with black race (*p* = .04), Table 3.

Adult IFG (88 yes, 617 no) was positively associated with adult BP and TG (high vs not high), *p* < .0001, *p* = .0009, respectively, and with cigarette smoking, *p* = .018, Table 3.

Neither pediatric nor young adult LDLC was associated with young adult CVD, p > .05.

## Discussion

In the current study, risk factors for CVD retained from childhood to adulthood predicted CVD in young adulthood. Risk for CVD was attenuated when childhood risk factors were not maintained into adulthood, congruent with the report by Juonala et al. [12]. Children who had high TG and retained high TG as adults had increased CVD events. Children who had normal BMI and retained normal BMI as adults had reduced CVD events. Children who had high childhood BP and TG and retained these into adulthood were more likely to have adult T2DM, children with childhood risk factors not retained were not associated with increased adult T2DM, congruent with the report by Juonala et al. [12].

In contrast to CVD and T2DM, adult IFG was associated with adult high BP, TG, and cigarette smoking, and was not associated with retention of risk factors from childhood to adulthood.

Our finding of a significant association of high TG retained from childhood to adulthood with young adult CVD is consistent with pediatric [8] and adult studies where non-fasting [23-25] and fasting TG [26-29] are independent risk factors for CVD and for ischemic stroke [30]. The association of TG high from childhood through young adulthood with adult CVD may, speculatively, reflect the presence of pediatric metabolic syndrome, a known predictor of adult CVD [18]. Moreover, TG levels in adolescent males have been related to coronary artery calcification 15 to 20 years later in young adults [31]. Coronary artery streaks in 6 to 30 year olds are significantly correlated with antecedent TG and very low density lipoprotein cholesterol [32,33]. In a post-mortem study of 15 to 34 year old men, the percentage of the right coronary arterial intima involved with atherosclerosis correlated with a combination of LDL and VLDL cholesterol levels, and was inversely associated with HDL cholesterol [34].

Normal childhood BMI retained to adulthood was a significant negative risk factor for adult CVD. The association of normal BMI retained from childhood to adulthood with low young adult CVD events is consistent with the report by Chen et al. [35] where clustering of bottom quartile BMI, HOMA IR, SBP, and the ratio of total/HDL cholesterol was associated with decreasing mean values of carotid intima-media thickness in adulthood.

High childhood BP and TG, two components of the metabolic syndrome complex, retained into adulthood were associated with adult T2DM, findings broadly in agreement with those of Everhart et al. [15] and Lee et al. [16], which suggested that the duration of the risk factor presence from childhood to young adulthood and the cumulative exposure to risk factors predict adult outcomes.

Our finding of an association of glucose levels in childhood with the development of T2DM in adulthood is consistent with a recent report from the Bogalusa Heart Study that fasting plasma glucose in childhood is a predictor of T2DM in young adulthood even when the pediatric glucose is within the normal range [36]. Moreover, childhood insulin response during an oral glucose challenge predicts adult acute insulin response [37].

Given the significant tracking of risk factors for CVD and T2DM as observed in the current and previous studies [11,38-40], failure to act on such childhood risk factors high TG, high BP, and obesity [12,16] means the underlying pathology may continue into young adulthood, increasing the likelihood of an adverse outcome [10,41]. These findings emphasize the importance of risk factor screening in childhood [1]. Lifestyle [1] and pharmacologic intervention [1,42,43] in childhood-adolescence might prevent development of CVD or T2DM in young adulthood.

A weakness in the current study is the absence of knowledge concerning when (at what age) participants with normal factors in childhood developed abnormal risk factors and when participants with abnormal factors in childhood developed normal risk factors. Thus, it was not possible to evaluate more precisely the length of time the at-risk state existed.

In the current study, neither pediatric nor young adult LDLC was associated with young adult CVD, perhaps attributable to treatment of high LDLC in 26 of 770 (3. 4%) young adults at PFS, or to the fact that with only 19 CVD endpoints by mean age 38, the study may not have had adequate power to declare an LDLC effect significant. Treatment to lower LDLC might, speculatively, also have reduced the power of LDLC to predict CVD.

Conventionally, parental history of CVD serves as an indication for screening for lipid abnormalities in children [44,45]. After detailed review of basing childhood screening on parental history, the recent Expert Panel statement [1] called for universal risk factor screening in children [1]. Identification of CVD risk factors in a child can directly facilitate primary prevention [1] in the child through young adulthood, and also focus diagnostic attention on the potentially high-risk parent.

## Conclusions

Risk factors for CVD and T2DM retained from childhood to adulthood predict CVD and T2DM in young adulthood and support universal childhood screening.

## Abbreviations

LRC: Lipid Research Clinics; PFS: Princeton School Follow-up Study; CVD: cardiovascular disease; CHD: coronary heart disease; CIMT: carotid intima-media thickening; IFG: impaired fasting glucose; T2DM: type 2 diabetes mellitus; HBP: high blood pressure; DBP: diastolic blood pressure; SBP: systolic blood pressure; TG: triglyceride; MetS: metabolic syndrome; HDLC: high density lipoprotein cholesterol; LDLC: low density lipoprotein cholesterol; BMI: body mass index; NCEP: National Cholesterol Education Program; NHLBI: National Heart, Lung, and Blood Institute; AHA: American Heart Association.

## Competing interests

The authors declare that they have no competing interests.

## Authors’ contributions

JAM and CJG designed the study. JAM supervised the initial study in children and the prospective follow-up study in young adulthood. CJG, JAM, and PW edited, analyzed, and assessed the data. PW provided the major biostatistical expertise, JAM, the major epidemiologic expertise. All authors read and approved the final manuscript.
